# Performance and Interaction Mechanisms of Bitumen Modified with Devulcanized Rubber-Recycled Polymer Composites

**DOI:** 10.3390/polym18202537

**Published:** 2026-10-15

**Authors:** Akkenzhe Bussurmanova, Anar Akkenzheyeva, Uzilkhan Yensegenova, Viktors Haritonovs, Gerda Eida, Aina Bernava, Romans Kornisovs, Yerzhan Imanbayev, Maira Turkmenbayeva, Paulius Urbonavicius, Murshida Aimova, Yerbolat Ayapbergenov, Martynas Jankauskas

**Affiliations:** 1 Pedagogy Faculty, Yessenov University, 32 Microdistrict, Aktau 130003, Kazakhstan; akkenzhe.bussurmanova@yu.edu.kz (A.B.); murshida.aimova@yu.edu.kz (M.A.); 2 Engineering Faculty, Yessenov University, 32 Microdistrict, Aktau 130003, Kazakhstan; anar.akkenzheyeva@yu.edu.kz (A.A.); maira.turkmenbayeva@yu.edu.kz (M.T.); 3 Faculty of Chemistry and Chemical Technology, Al-Farabi Kazakh National University, Al-Farabi Ave. 71, Almaty 050040, Kazakhstan; 4 Faculty of Civil and Mechanical Engineering, Riga Technical University, 6A Kipsalas Street, LV-1048 Riga, Latvia; viktors.haritonovs@rtu.lv (V.H.); romans.kornisovs@rtu.lv (R.K.); paulius.urbonavicius_1@edu.rtu.lv (P.U.); 5 Faculty of Natural Sciences and Technologies, Riga Technical University, 3 Paula Valdena Street, LV-1048 Riga, Latvia; gerda.eida@rtu.lv (G.E.); aina.bernava@rtu.lv (A.B.); 6 Laboratory of Petrochemical Processes, RSE “Institute of Combustion Problems”, Bogenbay Street, 172, Almaty 050012, Kazakhstan; erzhan.imanbayev@mail.ru; 7 Branch of LLP “KMG Engineering” “KazNIPImunaigas”, 35 Microdistrict, Section 6/1, Aktau 130000, Kazakhstan; ayapbergenov_e@kaznipi.kz; 8 JSC “Polylema”, Jegaines Str. 8, Kauno District, LT-54469 Biruliskes, Lithuania; info@polylema.lt

**Keywords:** devulcanized rubber, bitumen modification, recycled polymers, sustainable pavement materials, hybrid rubber–polymer modifiers

## Abstract

The accumulation of end-of-life tires and post-consumer polyolefin waste is a growing environmental burden, and their reuse as bitumen modifiers offers a sustainable, large-volume outlet in road construction. This study investigated paving-grade bitumen modified with four pre-compounded, partially devulcanized rubber (DVR)-recycled-polymer composites, differing in polymer type and, for two of the systems, in styrene–butadiene–styrene (SBS) chain architecture (linear or radial), added at 9–15 wt.%. The modified binders were characterized by penetration, softening point, elastic recovery, force ductility, low-temperature brittleness, and dynamic shear rheometry (DSR), together with FTIR, TGA, and fluorescence microscopy. All four composites improved binder stiffness, elasticity, and high-temperature rheology relative to the unmodified bitumen, with the two SBS-containing systems performing best overall. At the highest modifier content, the best-performing composite roughly halved the penetration and raised both the softening point and the higher critical temperature or rutting parameter by approximately 28–30 °C, while also markedly improving elastic recovery and low-temperature flexibility relative to the base bitumen. These findings indicate that SBS-containing devulcanized-rubber/recycled-polymer composites are promising sustainable modifiers for producing high-performance bituminous binders from waste-derived materials.

## 1. Introduction

Bitumen modification is one of the most effective approaches for improving the durability and performance of asphalt pavements, and polymeric, rubber-based, and hybrid modifiers are widely used to broaden the service temperature range of asphalt binders and enhance their engineering performance [1,2,3,4,5,6,7]. The rheological behavior of polymer-modified bitumen depends strongly on the type and compatibility of the polymer; for example, EVA blends with low-density polyethylene (LDPE) form a more homogeneous polymer-rich network than individual waste polymers, improving rheological properties, storage stability, and resistance to permanent deformation [8].

Waste-polymer and rubber utilization in bitumen has been widely studied with variable success depending on polymer type and compatibility [9,10,11]. Crumb rubber from end-of-life tires is among the most widely used secondary modifiers, and SBS has similarly been used to restore reclaimed asphalt pavement binders [12,13,14]. Phase instability and storage separation remain a major challenge for rubberized and polymer-modified binders: devulcanized crumb rubber alone forms a three-dimensional network within bitumen [15], and combining SBS with desulfurized crumb rubber further enhances this network and improves rheological and aging performance [16,17]. Numerous hybrid rubber–polymer systems—including SIS/CR, SBS/EVA/EPDM, CR/SBS/wax, and polyurethane/SBS combinations—have been shown to improve storage stability and performance through similar network-forming mechanisms [18,19,20,21,22,23,24,25,26,27,28]. Rubber–polymer composites more broadly have been investigated as a circular-economy route for recycling rubber and plastic waste [29,30,31,32,33]; bio-based and other alternative modifiers have also been explored [34,35,36,37].

Among recycled polyolefins, recycled polyethylene (rPE) and recycled polypropylene (rPP)—the two base recycled polymers used in the present study—are of particular relevance. rPE has shown potential for enhancing the high-temperature performance of asphalt binders [38,39], and compatibilized rPE/rPP blends have been shown to improve dispersion and stiffness when combined with bitumen [40]. rPP similarly increases binder stiffness through the formation of a rigid polymer-rich phase, although comparative studies indicate that crumb rubber alone provides greater heat-resistance and stability gains than PP-based modification [41]. However, rPE and rPP have generally been evaluated either alone or blended with virgin SBS, rather than in combination with partially devulcanized rubber (DVR), and the specific influence of SBS molecular architecture (linear versus radial) on rPP–DVR interactions has not been systematically examined.

We hypothesize that combining DVR with rPE or rPP, and further with linear or radial SBS, produces distinct polymer–rubber network structures that differentially govern the high- and low-temperature performance of the modified bitumen. Building on this hypothesis, and given the research gap identified above, the present study compares four DVR-based composite modifiers containing rPE, rPP, rPP–linear SBS (rPP-SBS-linear), and rPP–radial SBS (rPP-SBS-radial), combining conventional binder characterization (penetration, softening point, elastic recovery, force ductility, and Fraass breaking point) with sol/gel fraction analysis, FTIR, TGA, and fluorescence microscopy to relate modifier structure, morphology, and thermal behavior to mechanical and rheological performance. This study provides a comparative evaluation of the resulting trade-offs among high-temperature stiffness, elasticity, cohesion, and low-temperature flexibility using standard, well-established characterization methods, complementing the prior literature on DVR- and SBS-modified bitumen.

The technical roadmap of the study is as follows: (i) four DVR-based modifiers (DVR-rPE, DVR-rPP, DVR-rPP-SBS-linear, DVR-rPP-SBS-radial) were characterized by gel-fraction analysis, FTIR, and TGA; (ii) the modifiers were blended with B70/100 bitumen at 9, 12, and 15 wt.%; (iii) the modified binders were evaluated by penetration, softening point, elastic recovery, force ductility, Fraass breaking point, and dynamic shear rheometry; and fluorescence microscopy and (iv) the FTIR, TGA and microscopy results were combined with the mechanical and rheological data to propose an interaction mechanism between partially devulcanized rubber and SBS-containing recycled polymers.

## 2. Materials and Methods

### 2.1. Materials

Modified binders were prepared using B70/100-grade petroleum bitumen supplied by “CASPI BITUM” JV LLP (Aktau, Kazakhstan). The key physicochemical parameters of the petroleum road bitumen (grade B70/100) are summarized in Table 1.

Customized, partially devulcanized rubber (DVR)-based compounds, supplied by “Polylema” JSC (Kaunas, Lithuania), were used in this study (Table 2). The materials were produced using a devulcanization technology developed by Regen RBR Inc., which involves the application of a selective catalyst capable of cleaving sulfur cross-links at relatively low temperatures while preserving most of the polymer macromolecular structure [45]. The original rubber crumb employed for DVR production had a particle size of 0.08 mm.

During the production, rubber crumb was compounded with one of four polymer modifiers: recycled polyethylene (rPE), recycled polypropylene (rPP), recycled polypropylene—linear styrene–butadiene–styrene (rPP-SBS-linear), or recycled polypropylene—radial styrene–butadiene–styrene (rPP-SBS-radial). The resulting DVR-based modifiers were subsequently used for bitumen modification. The DVR-based modifiers were produced in the form of black granules. The composition of the modifiers depended on the type of recycled polymer used and is summarized as follows:

- DVR-rPE: 85 wt.% partially devulcanized rubber, 10 wt.% recycled polyethylene (rPE), and 5 wt.% additives.
- DVR-rPP: 85 wt.% partially devulcanized rubber, 10 wt.% recycled polypropylene (rPP), and 5 wt.% additives.
- DVR-rPP-SBS-linear: 64 wt.% partially devulcanized rubber, 9 wt.% recycled polypropylene (rPP), 22 wt.% linear styrene–butadiene–styrene (SBS), and 5 wt.% additives.
- DVR-rPP-SBS-radial: 65 wt.% partially devulcanized rubber, 10 wt.% recycled polypropylene (rPP), 20 wt.% radial styrene–butadiene–styrene (SBS), and 5 wt.% additives.

Two limitations of this formulation set should be noted. First, the DVR-rPP-SBS-linear and DVR-rPP-SBS-radial composites, which are compared throughout this study to demonstrate the effect of SBS molecular architecture, are commercial products that also differ slightly in DVR, rPP, and SBS content (linear: 9 wt.% rPP/22 wt.% SBS; radial: 10 wt.% rPP/20 wt.% SBS); consequently, the performance differences attributed to linear-versus-radial SBS architecture in Section 3.4, Section 3.5, Section 3.6, Section 3.7, Section 3.8 and Section 3.9 may also partly reflect this compositional difference, and the two effects cannot be fully separated with the present commercial formulations. Second, the average molecular weight and polydispersity of the rPE, rPP, and SBS components, the styrene content and branching degree of the two SBS grades, and the exact chemical composition of the 5 wt.% additive package are proprietary information held by the modifier supplier (“Polylema” JSC) and were not disclosed to the authors; this information is required for full reproducibility of the modifiers themselves (though not of the bitumen-modification and testing procedures described below) and should be requested from the supplier and reported in a future revision if it can be released.

### 2.2. Development of Modified Bitumen

**Figure 1:**
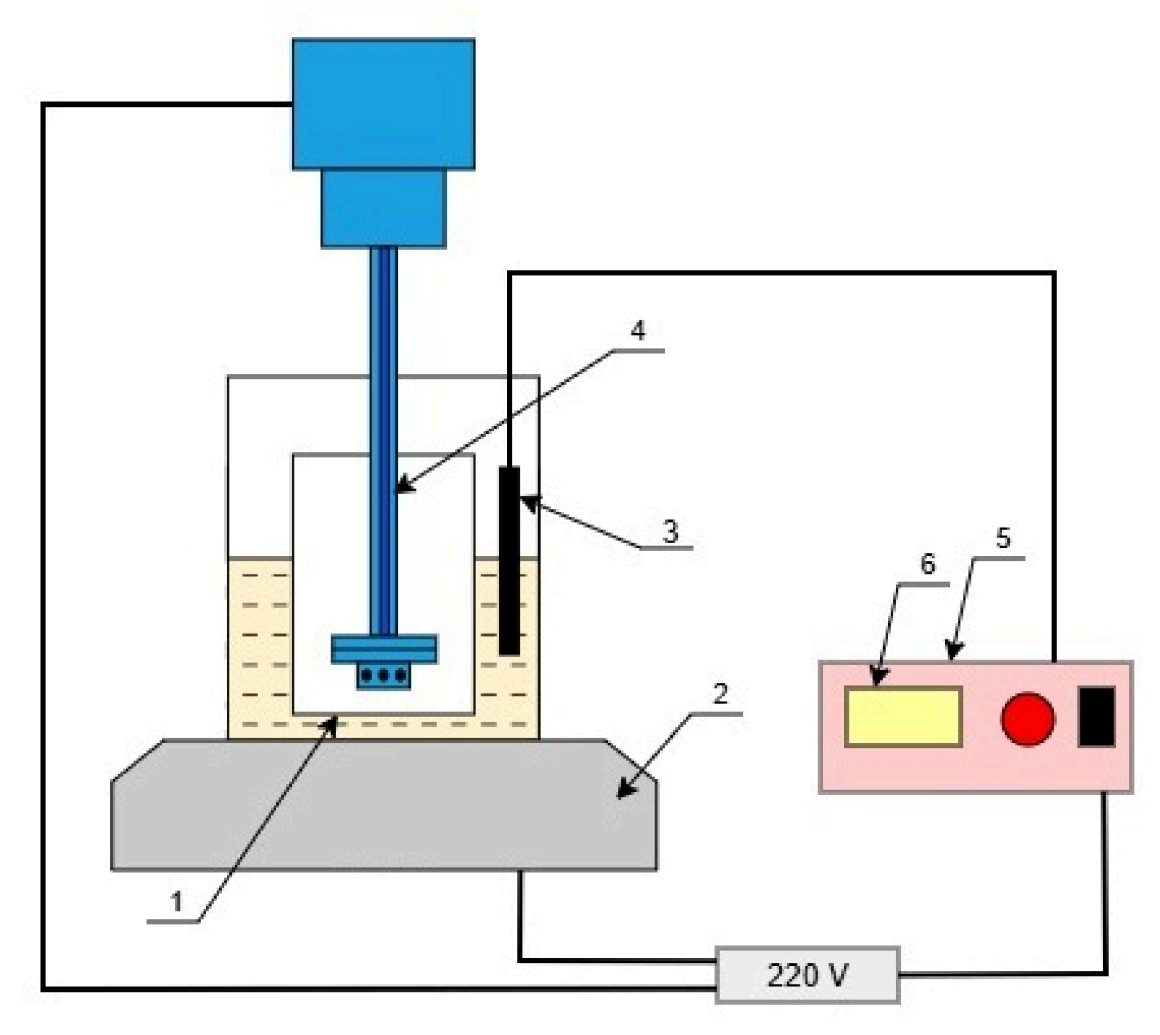
Schematic diagram of the experimental setup used for the preparation of modified bitumen binders: 1—reactor; 2—hot plate; 3—thermometer; 4—high-shear mixer; 5—high-shear mixer speed controller; 6—automatic temperature controller.

A laboratory-scale mixing system was assembled for the preparation of the modified bitumen samples. The schematic diagram of the setup is shown in Figure 1.

Modified bituminous binder was prepared using a high-shear mixer (model L5M-A, Silverson Machines Inc., East Longmeadow, MA, USA). Prior to blending, the base bitumen was heated to 140 °C and transferred into a mixing vessel placed in an oil bath. Subsequently, the DVR modifier was gradually added at dosage ranges of 9%, 12%, and 15% by weight of bitumen. The blending process was conducted at 180 °C and a rotational speed of 4500 rpm for 60 min to ensure uniform dispersion of the modifier within the bitumen matrix.

### 2.3. Characterization of Devulcanized Rubber

The penetration test was performed in accordance with EN 1426 to determine the consistency of the bituminous binders and evaluate their susceptibility to deformation under standardized loading conditions [42]. Measurements were carried out using a Matest B056-01 semi-automatic digital penetrometer (Matest S.p.A., Arcore, Italy). Three parallel measurements were made. Based on the original replicate measurements (now analyzed statistically; see Section 3.4 below), the average relative standard deviation across all measured concentrations and modifier systems was approximately 1.6% (range: 0.7–2.3%).

The softening point (Ring-and-Ball) test was conducted in accordance with EN 1427 to evaluate the temperature susceptibility of the binders and their resistance to elevated temperatures [43]. Two parallel measurements from the independent test specimens were performed for each sample using a Matest B070M Softmatic automatic Ring-and-Ball apparatus (Matest S.p.A., Arcore, Italy), with glycerol employed as the heating medium. The average standard deviation of the test results was ±2%.

The force ductility (cohesion) test was carried out in accordance with EN 13589 using standard dumbbell-shaped specimens [46]. Testing was performed with a Matest B055-20M asphalt ductility tester (Matest S.p.A., Arcore, Italy) at a temperature of 25 °C and a crosshead speed of 50 mm/min. Three parallel measurements from the independent test specimens were performed for each sample. During the test, the tensile force and specimen elongation were continuously recorded, and the deformation energy was determined as the area under the force–elongation curve.

Elastic recovery was determined in accordance with the ductilometer method described in EN 13398 [47], using the same Matest B055-20M asphalt ductility tester (Matest S.p.A., Arcore, Italy) employed for the force ductility test, at 25 °C. Three parallel measurements from the independent test specimens were performed for each sample. Each specimen was elongated to 200 mm at a crosshead speed of 50 mm/min, cut at mid-length, allowed to rest for 60 min, and the elastic recovery was calculated from the recovered length as a percentage of the initial 200 mm elongation.

Dynamic shear rheometry (DSR) (Anton Paar, Graz, Austria) was performed on the original binders in accordance with AASHTO T315, in oscillatory strain-controlled shear at a frequency of 10 rad/s (the standard AASHTO T315 test frequency), over a temperature sweep from 46 to 100 °C at 6 °C increments by using 25 mm parallel plates with a 1 mm gap for the entire 46–100 °C range. Two parallel measurements from the independent test specimens were performed for each sample.

Fourier-transform infrared (FTIR) spectra were recorded using an IRTracer-100 (Schimadzu Corporation, Kyoto, Japan) with an ATR accessory equipped with a diamond crystal in the wavenumber range of 4000–500 cm^−1^. Spectral measurements were performed using a PIKE MIRacle attenuated total reflectance (ATR) accessory equipped with a germanium crystal.

Thermal properties were investigated by thermogravimetric analysis (DTA/TGA) using a Mettler/Toledo 3+ (Mettler/Toledo, Columbus, OH, USA). The measurements were carried out under a nitrogen atmosphere with a gas flow rate of 50 mL/min. Alumina crucibles were used for all experiments. The samples were heated from room temperature to 1000 °C at a heating rate of 10 °C/min.

The gel fraction was determined by Soxhlet extraction in toluene. Approximately 1 g of each modifier sample was wrapped in a metallic sieve and bound with metallic wire together with an identification tag. Thus prepared, the test specimens were inserted into the Soxhlet apparatus, and solvent extraction was performed by refluxing in toluene for 72 h. The gel fraction (GF) was calculated as the percentage of remaining mass relative to the initial sample weight by considering inorganic filler content, which was determined by TGA. GF was determined in duplicate.

Fluorescence microscopy tests were performed using an optical microscope with an epifluorescence unit, a mercury lamp and a 480/30 nm excitation filter, and imaging was carried out with a Moticam ProS5 Lite system (Motic Europe S.L.U., Barcelona, Spain). Sample preparation and imaging conditions were kept strictly the same for all specimens so that the fluorescence observations remain comparable. The cross-sectional surface of each specimen was examined under fluorescence excitation to assess the spatial distribution and morphology of the fluorescent phases. Observations were conducted at 100× magnification.

Statistical analysis: comparative statements throughout Section 3 (e.g., “highest,” “significantly,” “most effective,” “closely comparable”) were assessed by OriginPro 2021 software (version 9.8; OriginLab Corp., Northampton, MA, USA). A two-way analysis of variance (modifier type × concentration) with Tukey’s HSD post hoc test (α = 0.05) was applied to the original replicate measurements for penetration, softening point, elastic recovery, force ductility, and Fraass breaking point. Gel fraction (duplicate measurements only, no concentration factor) data were assessed by one-way ANOVA with the same post hoc test. Both main effects and their interaction were statistically significant (*p* < 0.05) for all five properties analyzed by two-way ANOVA, whereas modifier type was significant for gel fraction (*p* < 0.01). The full ANOVA and Tukey HSD results are summarized where relevant in Section 3. Error bars in the figures represent ±1 standard deviation calculated from the original replicate measurements.

## 3. Results

### 3.1. Gel Fraction

**Figure 2:**
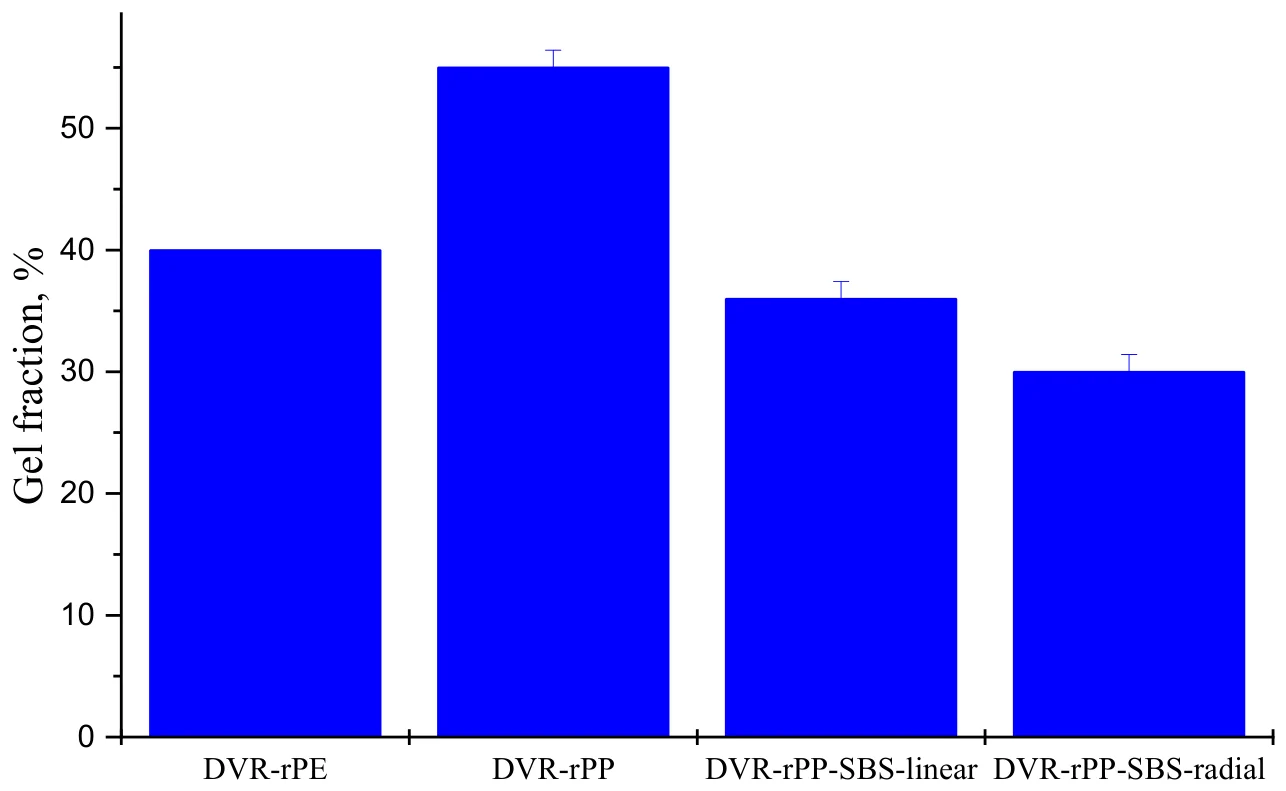
Gel fraction of the investigated bitumen modifiers.

GF results of the investigated specimens, summarized in Figure 2, provided information about the cross-linking degree of the material.

GF values have been corrected for inorganic filler content determined by TGA. The uncorrected GF values were 62%, 70%, 55%, and 53% for DVR-rPE, DVR-rPP, DVR-rPP-SBS-linear, and DVR-rPP-SBS-radial, respectively. All GF values reported in Section 3.1 and used in the statistical analysis below are the ash-corrected values. In comparison to tire rubber, which typically has a crosslinking degree around 90% and above, the measured GF values were considerably lower, indicating disruption of the 3D structure of the rubber due to partial devulcanization. During the devulcanization technology used, S–S bonds were predominantly broken due to their lower activation energy compared with C–S and C–C bonds. The highest GF value among the investigated rubber modifiers was obtained for DVR-rPP, followed by DVR-rPE and by DVR-rPP-SBS-linear and DVR-rPP-SBS-radial. The lower GF of the SBS-containing composites is most likely due to lower rubber content in the compositions.

One-way ANOVA confirmed a statistically significant effect of modifier type on GF (F(3,4) = 151.4, *p* = 1.4 × 10^−4^). Tukey’s HSD post hoc test showed that DVR-rPP-SBS-radial differed significantly from all three other systems (*p* ≤ 0.03) and that DVR-rPP differed significantly from DVR-rPE (*p* < 0.001); the difference between DVR-rPP-SBS-linear and DVR-rPE was not statistically significant (*p* = 0.10), indicating that these two systems had comparable GF despite their different polymer compositions.

### 3.2. FTIR Characterization of DVR-Based Modifiers

**Figure 3:**
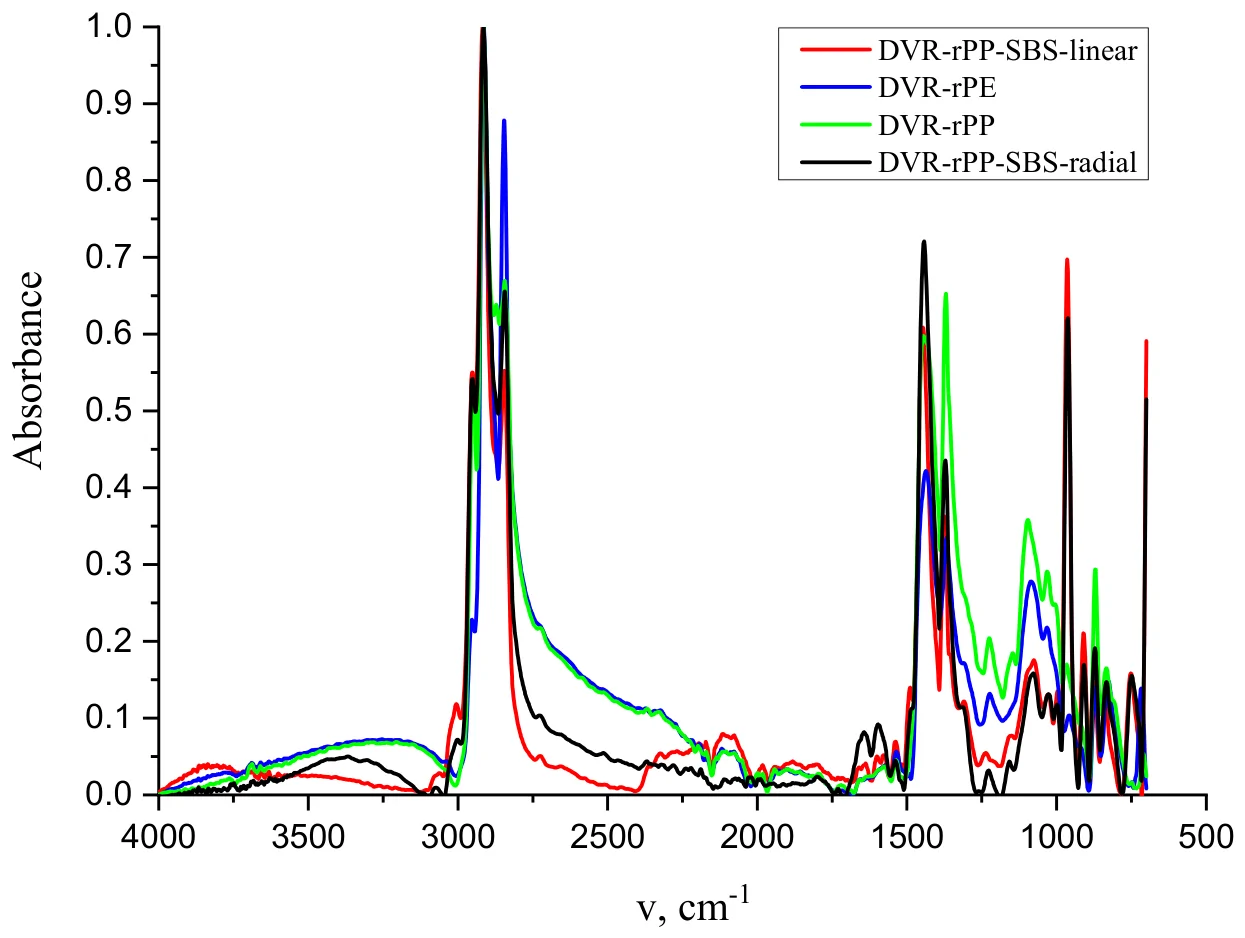
The infrared spectra of the investigated bitumen modifiers: DVR-rPE, DVR-rPP, DVR-rPP-SBS-linear, and DVR-rPP-SBS-radial.

Fourier-transform infrared spectroscopy (FTIR) was employed to investigate the chemical structure of the DVR-based modifiers and to evaluate the effects of polymer composition and partial devulcanization on the functional groups present in the materials. The FTIR spectra of DVR-rPE, DVR-rPP, DVR-rPP-SBS-linear, and DVR-rPP-SBS-radial are presented in Figure 3.

The FTIR spectrum of DVR-rPE exhibited strong absorption peaks at approximately 2950, 2921, and 2850 cm^−1^, corresponding to asymmetric and symmetric C–H stretching of aliphatic CH_2_/CH_3_ groups typical of polyisoprene, polybutadiene, and polyolefin structures in waste tire rubber and recycled polyethylene, consistent with previous reports on crumb-rubber-modified asphalt and FTIR studies of devulcanized rubber products [48,49,50]. The band near 1450 cm^−1^ was assigned to CH_2_/CH_3_ bending vibrations. A distinct peak at ≈1080 cm^−1^, attributed to sulfoxide (S=O) groups formed during devulcanization [51], and a peak at 966 cm^−1^, corresponding to C=C vibrations of polybutadiene segments, indicated that a significant fraction of the elastomeric backbone remained intact after devulcanization, while additional bands near 910 cm^−1^ (terminal vinyl groups from chain scission), 873/812 cm^−1^ (substituted aromatic rings from styrene-containing rubbers), and 699 cm^−1^ (styrene-containing structures, also seen for SBR) confirmed that the basic polymer framework was largely preserved. An additional band at 721 cm^−1^, corresponding to long-chain methylene sequences characteristic of polyethylene, confirmed the incorporation of recycled polyethylene in DVR-rPE.

DVR-rPP showed similar bands, confirming that the devulcanized-rubber phase dominates the spectral profile, with only minor intensity differences from DVR-rPE in the 2800–3000, 1300–1500, and 650–750 cm^−1^ regions reflecting differences in polymer composition. The spectra of DVR-rPP-SBS-linear and DVR-rPP-SBS-radial displayed additional SBS-related bands together with those of the devulcanized rubber, along with a reduced intensity of the 1080 cm^−1^ (S=O) band; this intensity change should be interpreted cautiously, since it may reflect compositional or normalization differences rather than a direct measure of interaction strength, and is consistent with, but does not on its own establish, closer physical association between SBS and the devulcanized rubber [52,53,54].

Overall, the FTIR analysis indicates that the primary rubber chain structure was partly preserved during devulcanization, complying with GF results. The FTIR spectra also confirm the presence of the expected functional groups from the recycled polyolefins and SBS in the corresponding DVR-based composites. This chemical evidence reflects functional-group composition rather than phase compatibility or network continuity as such, so the superior mechanical and rheological performance of the SBS-containing systems (Section 3.4, Section 3.5, Section 3.6, Section 3.7, Section 3.8, Section 3.9 and Section 3.10) is interpreted together with the mechanical, rheological, and fluorescence-microscopy results rather than from the FTIR data alone.

### 3.3. Thermal Stability of DVR-Based Modifiers

**Figure 4:**
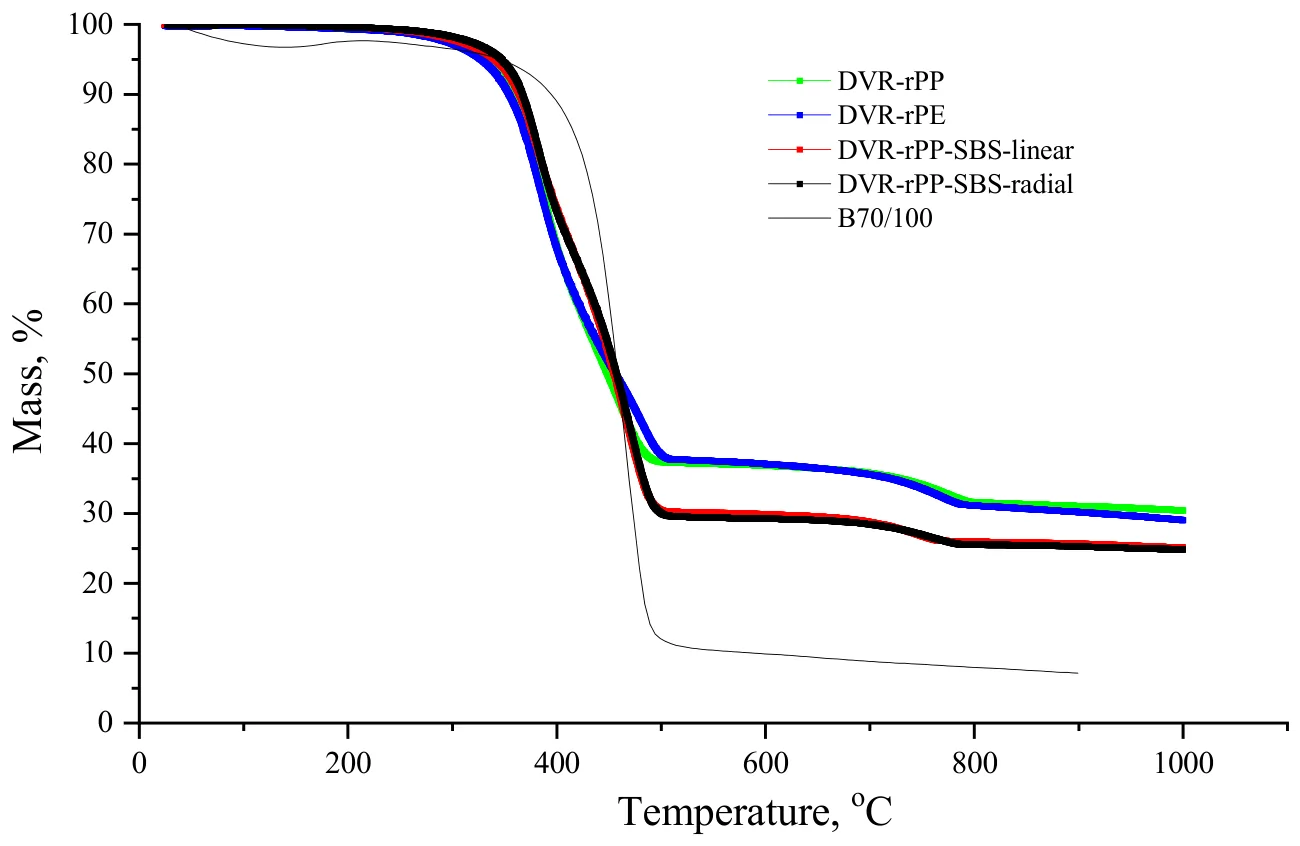
TG curves of DVR-rPE, DVR-rPP, DVR-rPP-SBS-linear, DVR-rPP-SBS-radial, and B70/100 recorded under a nitrogen atmosphere.

The thermal stability and decomposition behavior of the DVR-based modifiers and 70/100 bitumen were evaluated by thermogravimetric and differential thermal analysis (TGA/DTA) under a nitrogen atmosphere. In Figure 4, the thermogravimetric profile of neat 70/100 bitumen is depicted, revealing a two-stage decomposition pattern with major weight loss within the temperature regions of ≈350 °C and ≈500 °C, accompanied by minor weight change at relatively low temperatures until 200 °C, which most likely is related to the evolution of the bitumen light fractions.

In Figure 4, the TGA/DTA curves of DVR-rPE, DVR-rPP, DVR-rPP-SBS-linear, and DVR-rPP-SBS-radial modifiers are also presented. The combined representation enables direct comparison of the thermal degradation profiles of the four DVR-based systems and provides insight into the influence of recycled polyolefins and SBS on their thermal behavior.

All four DVR-based modifiers exhibited multistage thermal degradation, characteristic of multicomponent systems and consistent with reports for ground tire rubber, devulcanized rubber, and SBR/PP- and SBS/crumb-rubber composites [26,49,50,51,52]; in all cases, major mass loss occurred well above the 180 °C processing temperature used for bitumen modification. DVR-rPE showed a primary mass-loss stage of ≈62.8 wt.% between 350 and 500 °C, a secondary loss of ≈6.3 wt.%, and a residue of ≈29 wt.% at 1000 °C, consistent with previous studies on waste-polyethylene-modified asphalt [55]. DVR-rPP showed a broadly similar profile (≈63.3 wt.% between 350 and 600 °C, ≈5.4 wt.% secondary, ≈30 wt.% residue), with no substantial mass loss below ≈300 °C, indicating adequate thermal integrity for bitumen processing. The two SBS-containing systems showed a distinctly larger primary mass loss (>70 wt.%, mainly between 300 and 500 °C), a smaller secondary loss (≈3.6–3.8 wt.%), and a lower residue (≈25 wt.%) than DVR-rPE and DVR-rPP; despite their different SBS architectures, DVR-rPP-SBS-linear and DVR-rPP-SBS-radial displayed similar overall mass-loss profiles, consistent with reports that the thermal response of hybrid rubber/polymer systems depends primarily on the formation and interaction of the polymer-rich and rubber phases rather than on polymer architecture alone [52,56,57].

These differences should not be read as a simple ranking of thermal stability, since the final residue also reflects the relative content of polymeric material, carbon black, and mineral fillers; rather, they confirm clear compositional differences among the four modifiers, which may also translate into the distinct rheological behavior reported in Section 3.4, Section 3.5, Section 3.6, Section 3.7, Section 3.8, Section 3.9 and Section 3.10, given the close link between polymer network structure and rheological performance in SBS-modified bitumens [53]. From a processing perspective, all four modifiers remained thermally stable well above the 180 °C mixing temperature used in this study, with the onset of major degradation at least ≈120–150 °C higher, providing an adequate thermal margin during high-shear mixing and consistent with general recommendations for polymer-modified bitumen processing [54].

### 3.4. Effect of DVR-Based Modifiers on Penetration

**Figure 5:**
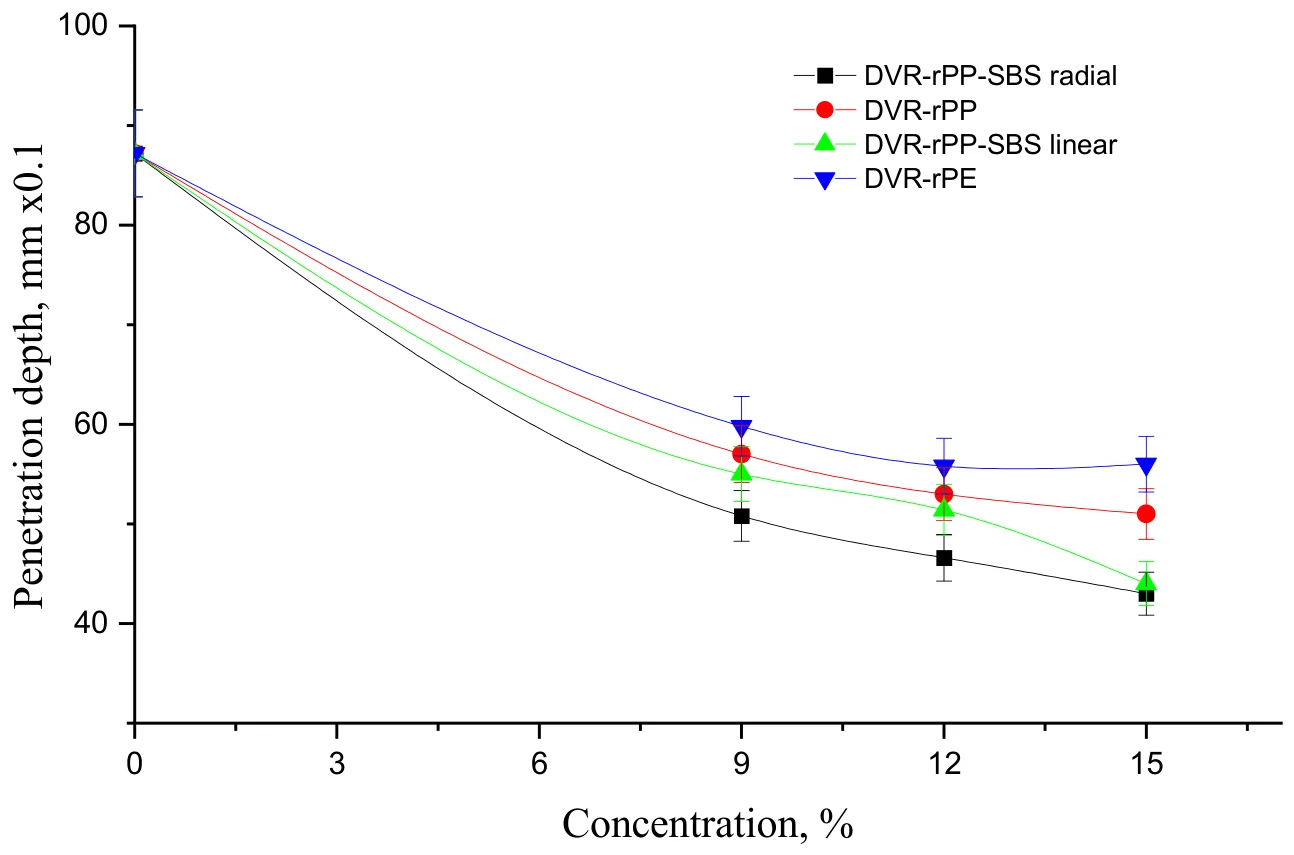
Penetration depth of B70/100 bitumen modified with DVR-rPP, DVR-rPP-SBS-linear, DVR-rPP-SBS-radial, and DVR-rPE as a function of modifier concentration.

The penetration values of the modified binders are presented in Figure 5.

The incorporation of DVR-based modifiers reduced the penetration of the base B70/100 bitumen at all concentrations, reflecting increased binder consistency and stiffness [1,13,54]. The reduction depended on both modifier concentration and polymer composition. DVR-rPP-SBS-radial produced the strongest stiffening effect, with penetration decreasing from 50.8 (0.1 mm) at 9 wt.% to 43.0 (0.1 mm) at 15 wt.%, closely followed by DVR-rPP-SBS-linear (55.0 to 44.0 (0.1 mm) over the same concentration range of the modifier. DVR-rPE decreased to 59.8 (0.1 mm) at 9 wt.% and then plateaued near 56 (0.1 mm) at 12–15 wt.%, while DVR-rPP showed a moderate overall reduction, from 57.0 to 51.0 (0.1 mm).

These trends indicate that the two SBS-containing systems developed the most effective polymer–rubber network, while DVR-rPP and DVR-rPE showed progressively more limited stiffening [9,39,52,53,54,55]; the composition- and architecture-dependent mechanistic basis for this ranking, common to all five properties examined in Section 3.4, Section 3.5, Section 3.6, Section 3.7 and Section 3.8, is discussed jointly in Section 3.9 rather than repeated for each property. These results are consistent with our previous study on rubber–polymer composites for bitumen modification [29].

Two-way ANOVA confirmed statistically significant effects of modifier type (F(3,24) = 249.4, *p* = 3.2 × 10^−18^), concentration (F(2,24) = 212.2, *p* = 5.5 × 10^−16^), and their interaction (F(6,24) = 13.6, *p* = 1.1 × 10^−6^) on penetration. Tukey’s HSD post hoc test showed that all six pairwise comparisons among the four modifier systems, including DVR-rPP-SBS-linear versus DVR-rPP-SBS-radial, were statistically significant (*p* < 0.05), confirming that penetration differed measurably among all four systems rather than only between broad groups.

### 3.5. Softening Point Characteristics

**Figure 6:**
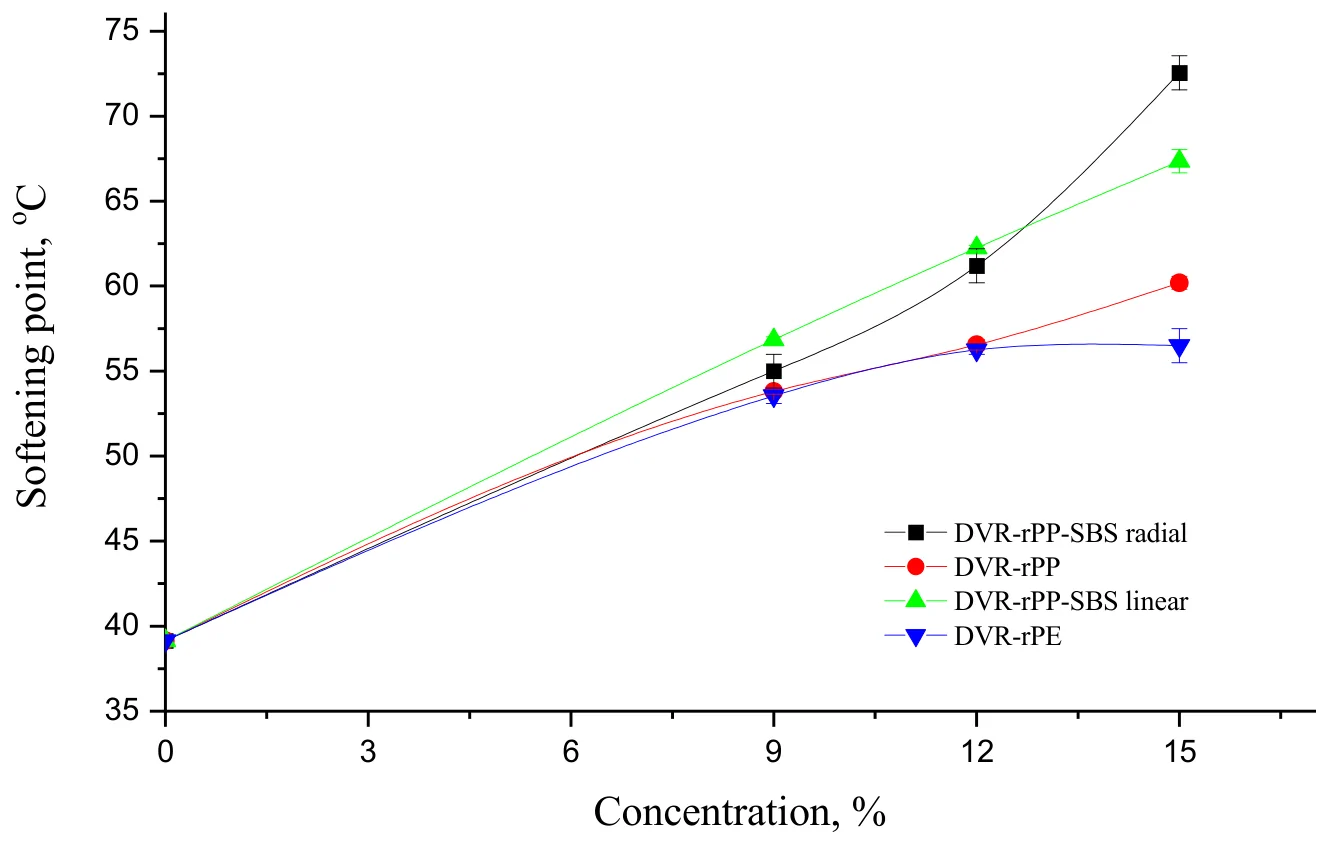
Softening point of B70/100 bitumen modified with DVR-rPP, DVR-rPP-SBS-linear, DVR-rPP-SBS-radial, and DVR-rPE as a function of modifier concentration.

The influence of DVR-based modifiers on the softening point of B70/100 bitumen is shown in Figure 6.

All DVR-based modifiers increased the softening point of the base bitumen, indicating improved resistance to flow at elevated temperatures [48], though the magnitude depended on both concentration and polymer composition. Both SBS-containing systems produced the greatest increases: DVR-rPP-SBS-linear rose fastest at 9–12 wt.%, reaching a total increase of approximately 28–30 °C at 15 wt.%, while DVR-rPP-SBS-radial, initially close to DVR-rPP-SBS-linear, increased more sharply between 12 and 15 wt.% and ultimately achieved the highest softening point of all systems (a total increase of approximately 33–34 °C). This contrasting behavior between the two SBS architectures is discussed jointly with the other properties in Section 3.9 (see also the compositional caveat in Section 2.1) [1,7,52,53,54]. DVR-rPP produced a progressive increase that exceeded DVR-rPE at 15 wt.%, consistent with its rigid, polymer-rich phase [9,41], while DVR-rPE showed the smallest, plateauing increase and was the least effective modifier at 15 wt.%, consistent with the more limited network-forming ability of polyethylene [8,39,55].

Overall, the polymer composition of the DVR composite, rather than DVR concentration alone, determined the high-temperature response: DVR-rPP-SBS-radial was most effective at 15 wt.%, followed by DVR-rPP-SBS-linear and DVR-rPP, while DVR-rPE was least effective. These trends are consistent with previous studies on polymer type, concentration, and phase morphology in polymer-modified bitumen [1,7,52,54] and with our previous study on DVR-based rubber–polymer composites [29].

Two-way ANOVA confirmed statistically significant effects of modifier type (F(3,24) = 280.1, *p* = 8.4 × 10^−19^), concentration (F(2,24) = 581.9, *p* = 4.6 × 10^−21^), and their interaction (F(6,24) = 65.6, *p* = 1.1 × 10^−13^) on softening point. Unlike the other properties examined, Tukey’s HSD post hoc test showed that DVR-rPP-SBS-radial and DVR-rPP-SBS-linear did not differ significantly from each other (*p* = 0.41), confirming that these two systems were statistically comparable for this property, while all other pairwise comparisons were significant (*p* < 0.05).

### 3.6. Elastic Recovery Performance

**Figure 7:**
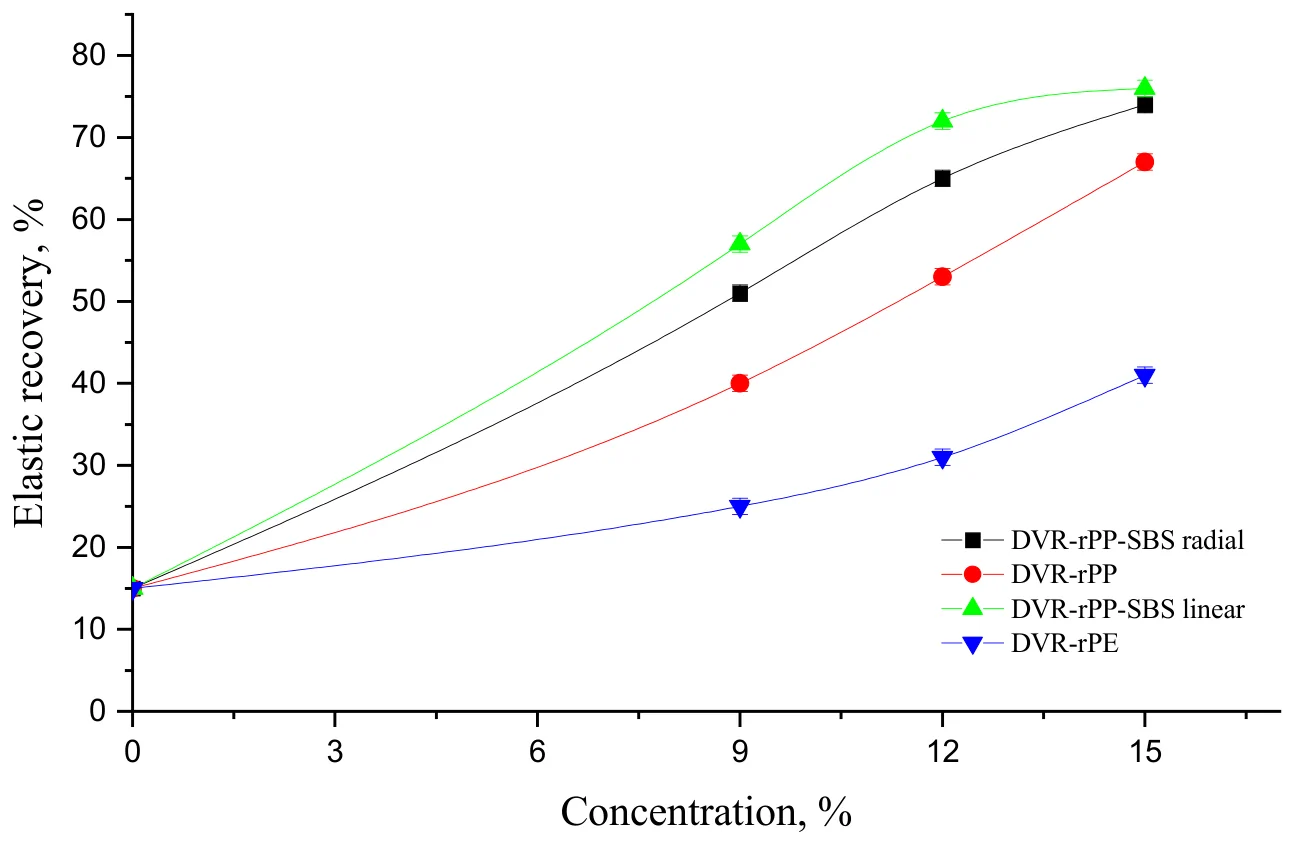
Elastic recovery of B70/100 bitumen modified with DVR-rPP, DVR-rPP-SBS-linear, DVR-rPP-SBS-radial, and DVR-rPE as a function of modifier concentration.

Elastic recovery is an important indicator of the ability of a bituminous binder to recover after deformation and is closely associated with its capacity to resist permanent deformation under repeated loading. The elastic recovery values of B70/100 bitumen modified with the four DVR-based systems are presented in Figure 7.

All modifiers increased elastic recovery relative to the base binder, consistent with rubber–polymer network formation reported for ground-tire-rubber-modified bitumens [48]. DVR-rPP-SBS-linear showed the most pronounced improvement, rising from ≈57% at 9 wt.% to ≈73% at 12 wt.% and plateauing near ≈76% at 15 wt.%, consistent with a well-developed elastomeric network already formed by 12 wt.% [1,7,16,53,54]. DVR-rPP-SBS-radial increased more gradually, from approximately 52% at 9 wt.% to 65% at 12 wt.% and 74% at 15 wt.%, converging with DVR-rPP-SBS-linear at 15 wt.%, consistent with the architecture-dependent network formation discussed in Section 3.9 [1,21,54]. DVR-rPP rose steadily from ≈40% to ≈67%, remaining below both SBS systems but showing accelerating improvement, consistent with its plastomeric character combined with the devulcanized-rubber phase [1,9,41]. DVR-rPE gave the lowest recovery throughout, rising from approximately 26% at 9 wt.% to 32% at 12 wt.% and 41% at 15 wt.%, consistent with the limited network-forming ability of polyethylene [39,56].

Overall, modifier composition had a stronger influence on elastic recovery: both SBS-containing systems reached the highest, closely comparable recovery at 15 wt.%, DVR-rPP approached but did not surpass them, and DVR-rPE remained lowest throughout. These trends agree with previous studies on polymer swelling, phase morphology, and network development in rubber- and polymer-modified binders [1,13,53,54] and with our previous study on DVR-based rubber–polymer composites [29].

Two-way ANOVA confirmed statistically significant effects of modifier type (F(3,24) = 2286, *p* = 1.3 × 10^−29^), concentration (F(2,24) = 1362.3, *p* = 2.0 × 10^−25^), and their interaction (F(6,24) = 28.3, *p* = 9.5 × 10^−10^) on elastic recovery. Tukey’s HSD post hoc test showed that all six pairwise comparisons among the four modifier systems, including DVR-rPP-SBS-linear versus DVR-rPP-SBS-radial, were statistically significant (*p* < 0.05).

### 3.7. Force Ductility Analysis

**Figure 8:**
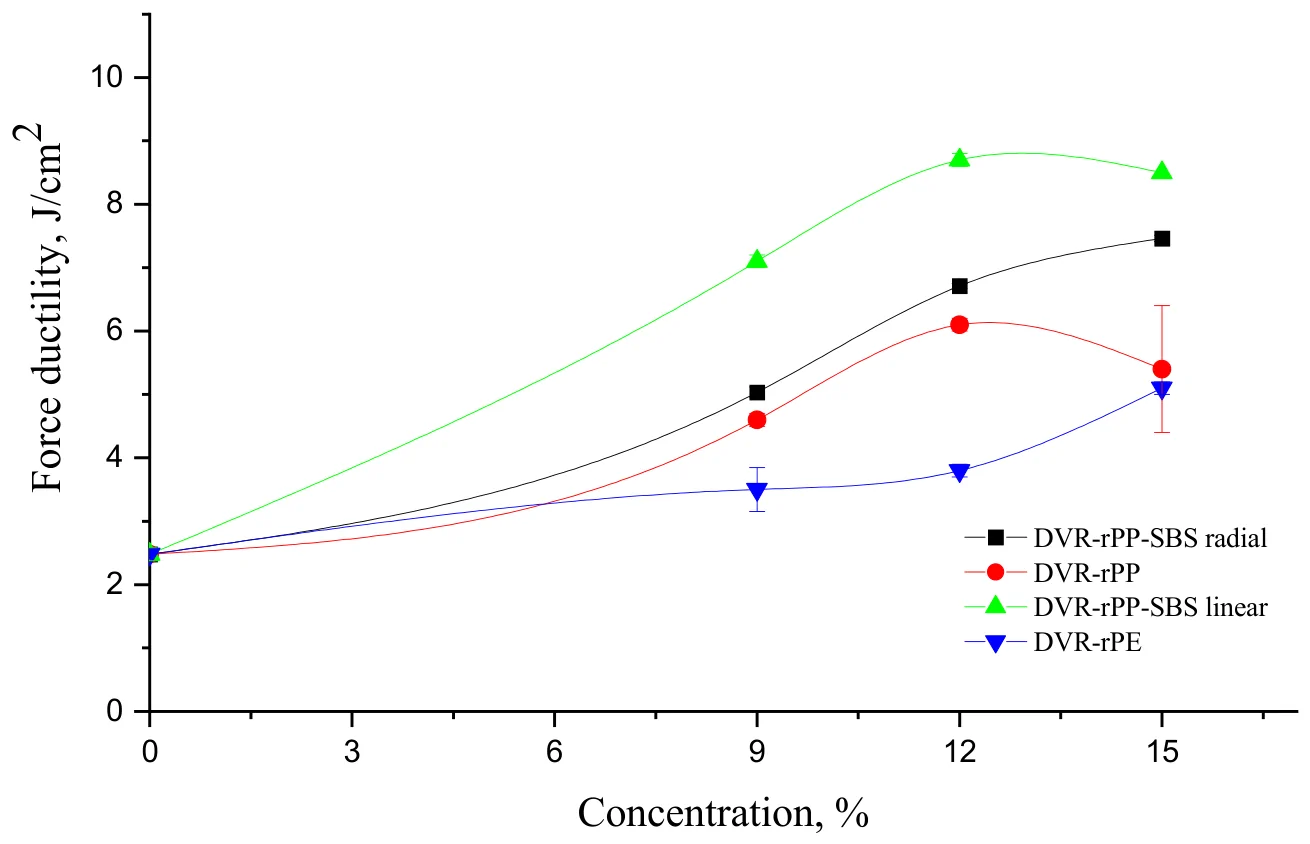
Force ductility of B70/100 bitumen modified with DVR-rPP, DVR-rPP-SBS-linear, DVR-rPP-SBS-radial, and DVR-rPE as a function of modifier concentration.

The force ductility results are presented in Figure 8. Force ductility characterizes the cohesive behavior of a modified binder and its ability to absorb deformation energy before failure. All the investigated DVR-based modifiers increased the deformation energy relative to the base B70/100 bitumen, although the magnitude and concentration dependence of this improvement varied substantially with the composition. This behavior is consistent with previous observations on ground-tire-rubber-modified bitumens, where the development of an interconnected rubber–polymer network was directly linked to enhanced deformation-energy absorption [48].

DVR-rPP-SBS-linear exhibited the highest deformation energy throughout, rising from ≈7.1 J/cm^2^ at 9 wt.% to ≈8.7 J/cm^2^ at 12 wt.% and plateauing near ≈8.6 J/cm^2^ at 15 wt.%, consistent with an interconnected elastomeric phase capable of redistributing stress [1,7,16,53]. DVR-rPP-SBS-radial increased progressively, from approximately 5.1 J/cm^2^ at 9 wt.% to 7.5 J/cm^2^ at 15 wt.%, without plateauing, indicating that its branched SBS network needed a higher polymer content to reach full load-bearing capacity [1,21,52,53]. DVR-rPP showed a non-monotonic response, consistent with its plastomeric character and a possible change in phase morphology at higher content [1,9,41]. DVR-rPE gave the lowest values throughout, rising from approximately 3.9 J/cm^2^ at 9 wt.% to 4.1 J/cm^2^ at 12 wt.% and 5.1 J/cm^2^ at 15 wt.%, consistent with the limited energy-dissipation capacity of polyethylene [39,55].

Two-way ANOVA confirmed statistically significant effects of modifier type (F(3,24) = 253.5, *p* = 2.7 × 10^−18^), concentration (F(2,24) = 82.4, *p* = 1.8 × 10^−11^), and their interaction (F(6,24) = 8.5, *p* = 5.4 × 10^−5^) on force ductility. Tukey’s HSD post hoc test showed that all six pairwise comparisons among the four modifier systems, including DVR-rPP-SBS-linear versus DVR-rPP-SBS-radial, were statistically significant (*p* < 0.05).

### 3.8. Low-Temperature Performance: Fraass Breaking Point

**Figure 9:**
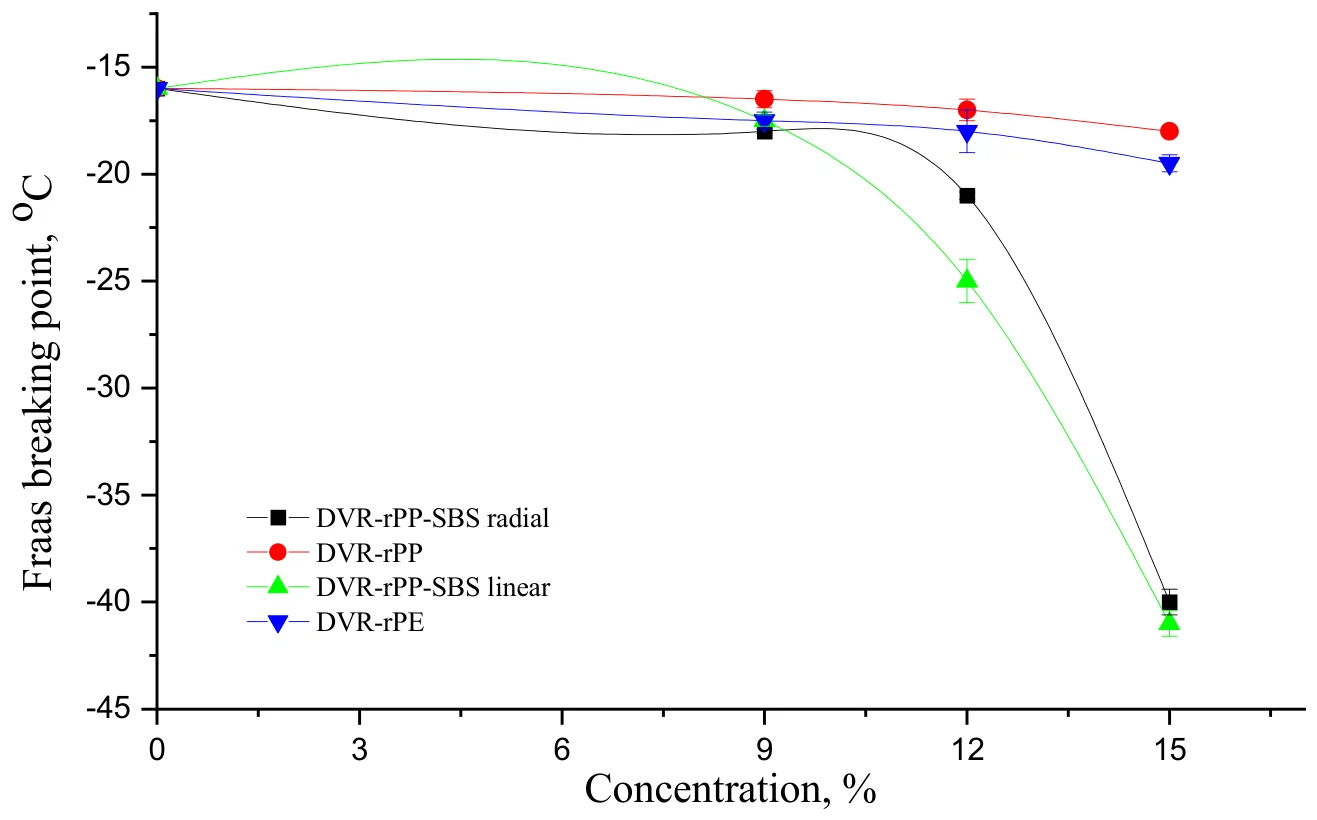
Fraass breaking point of B70/100 bitumen modified with DVR-rPP, DVR-rPP-SBS-linear, DVR-rPP-SBS-radial, and DVR-rPE as a function of modifier concentration.

The Fraass breaking point results are presented in Figure 9. The investigated DVR-based modifiers exhibited distinctly different effects on the low-temperature behavior of B70/100 bitumen, demonstrating that the resistance to thermal cracking depends not only on modifier concentration but also strongly on the composition of the polymer–rubber system. A shift in the Fraass breaking point toward lower temperatures indicates improved flexibility and a reduced susceptibility of the binder to brittle failure under low-temperature conditions.

DVR-rPP-SBS-linear produced the most pronounced improvement, with the Fraass breaking point decreasing from ≈−18 °C at 9 wt.% to ≈−25 °C at 12 wt.% and ≈−41 °C at 15 wt.%—the lowest of all systems, a ≈25 °C reduction from the base bitumen (−16 °C)—consistent with the network-forming mechanism discussed in Section 3.9 and by other authors [7,19,53,54]. DVR-rPP-SBS-radial followed a similar trend, reaching approximately −18 °C at 9 wt.%, −21 °C at 12 wt.%, and −37 °C at 15 wt.%, slightly outperforming the linear system at 9 wt.% but developing a less pronounced effect at higher content, remaining ≈3–4 °C above DVR-rPP-SBS-linear at 15 wt.% [1,48,52,54]. DVR-rPE showed an intermediate, modest improvement, from approximately −17.5 °C at 9 wt.% to −18 °C at 12 wt.% and −19 °C at 15 wt.%, consistent with the restricted low-temperature mobility of its crystalline plastomeric structure [39,55], while DVR-rPP showed the smallest improvement, from approximately −16 °C at 9 wt.% to −18 °C at 15 wt.%, remaining close to the base binder, consistent with the limited stress-relaxation contribution of polypropylene [9,41].

Overall, the low-temperature response reflected a balance between polymer-induced stiffening and elastomeric flexibility: DVR-rPP-SBS-linear gave the most favorable balance, DVR-rPP-SBS-radial a closely comparable one, DVR-rPE a moderate improvement, and DVR-rPP the least. This is consistent with rubber swelling and elastomeric network formation reported for SBS- and rubber-modified binders [13,52,53,54] and with our previous study on DVR-based rubber–polymer composites [29].

Two-way ANOVA confirmed statistically significant effects of modifier type (F(3,24) = 933.9, *p* = 5.7 × 10^−25^), concentration (F(2,24) = 1634.2, *p* = 2.3 × 10^−26^), and their interaction (F(6,24) = 415.2, *p* = 5.1 × 10^−23^) on the Fraass breaking point. Tukey’s HSD post hoc test showed that all six pairwise comparisons among the four modifier systems, including DVR-rPP-SBS-linear versus DVR-rPP-SBS-radial, were statistically significant (*p* < 0.05).

### 3.9. Dynamic Shear Rheometer (DSR) Analysis

**Figure 10:**
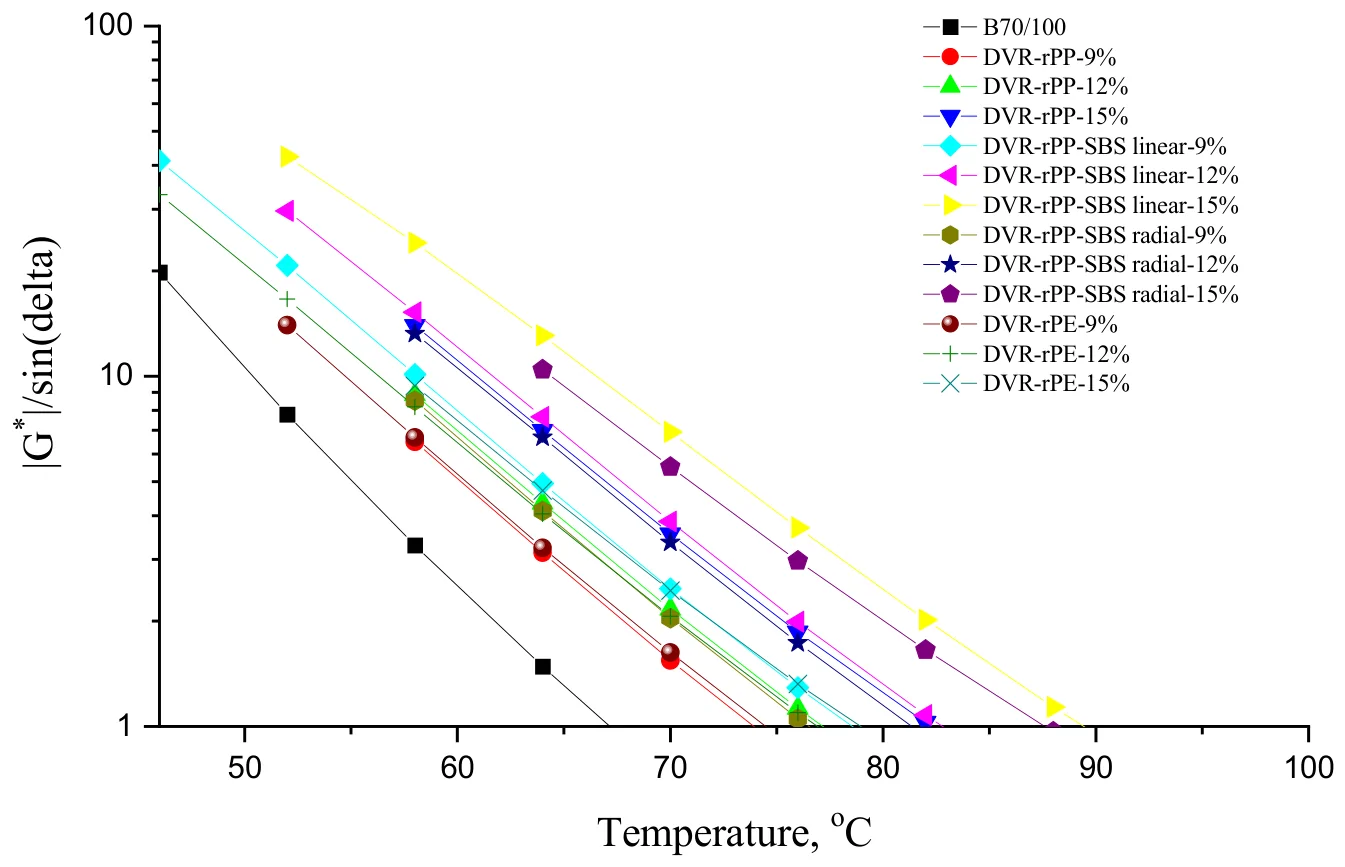
Rutting parameter |G*|/sin(δ) (DSR, original binder) of B70/100 bitumen modified with DVR-rPP, DVR-rPP-SBS-linear, DVR-rPP-SBS-radial, and DVR-rPE (9, 12, and 15 wt.%) as a function of temperature.

The higher critical temperature or rutting parameter |G*|/sin(δ), obtained by DSR on the investigated binders, is presented in Figure 10 as a function of temperature over the 46–100 °C temperature sweep [57]. For all systems investigated, |G*|/sin(δ) decreased monotonically with increasing temperature, reflecting the characteristic thermorheological softening of the bituminous binder.

The unmodified B70/100 bitumen exhibited the lowest |G*|/sin(δ) values throughout the tested range, decreasing from approximately 20 kPa at 46 °C to below the 1.00 kPa failure criterion defined by the Superpave specification [58] already within the 58–64 °C interval.

All DVR-based modifiers shifted the rutting curves upward and extended the temperature at which |G*|/sin(δ) fell below the 1.00 kPa criterion. The most pronounced improvement was obtained for DVR-rPP-SBS-linear-15%, which remained above the other systems across most of the 52–88 °C range (reaching approximately 42 kPa at 52 °C) and crossed the 1.00 kPa threshold only between 89 and 94 °C, an increase of approximately 28–30 °C relative to the base bitumen, comparable to the improvements reported for other well-structured SBS/rubber-modified binders [1,52]. DVR-rPP-SBS-radial-15% showed the second-highest value of 87 °C, remaining slightly below DVR-rPP-SBS-linear-15% at all higher temperatures.

DVR-rPE-12% displayed a notably high value (approximately 32 kPa), second only to DVR-rPP-SBS-linear-9% (approximately 41 kPa); however, this advantage narrowed rapidly with increasing temperature, and by 58 °C DVR-rPE-12% had fallen within the intermediate cluster of curves. This behavior is consistent with the plastomeric character of recycled polyethylene, which increases low-temperature stiffness but provides a comparatively limited improvement at higher service temperatures [39,55].

DVR-rPP-SBS-linear-12% and DVR-rPP-SBS-radial-9% exhibited lower |G*|/sin(δ) values than would be expected from interpolation between the neighboring concentrations of the same system, indicating a non-monotonic concentration dependence broadly similar to that previously observed for these formulations in the force ductility and Fraass breaking point analyses.

### 3.10. Fluorescence Microscopy Analysis

Fluorescence microscopy is widely used to visualize the distribution and swelling behavior of the polymer- and rubber-rich phase within polymer- and rubber-modified bitumen, since the aromatic-rich, swollen polymer/rubber particles fluoresce strongly under UV/blue excitation while the surrounding asphaltene-rich bitumen matrix remains comparatively dark [1,19]. This contrast enables direct visualization of particle size, swelling behavior, and the extent to which the modifier phase forms a continuous, interconnected structure within the bitumen matrix, providing microstructural information that complements the rheological and mechanical properties discussed in the preceding sections [13,53].

**Figure 11:**
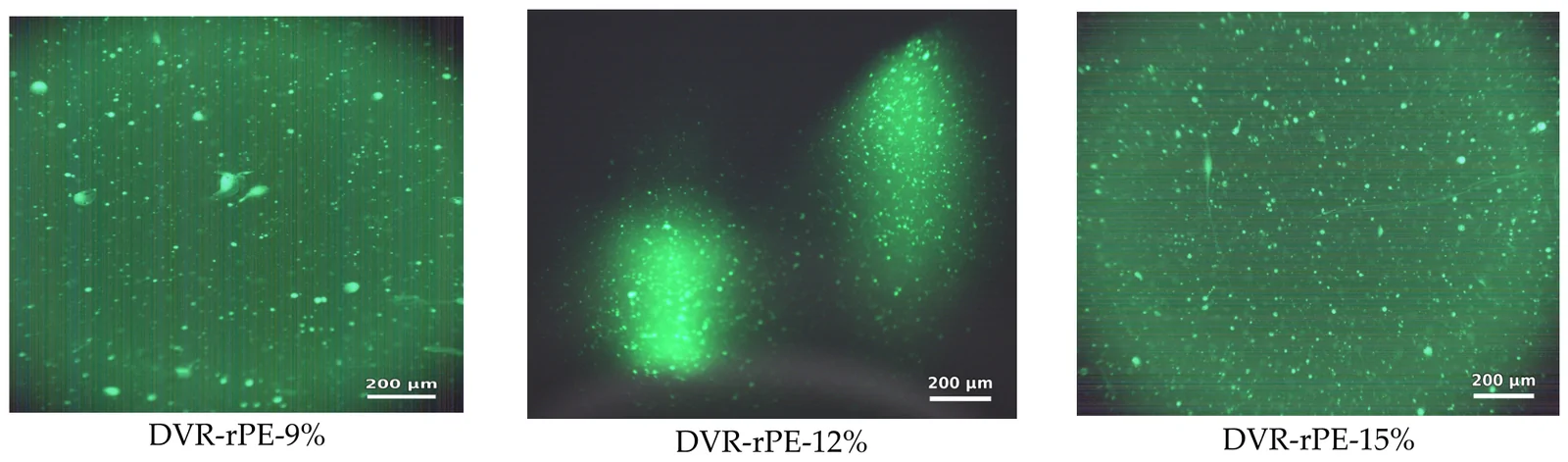

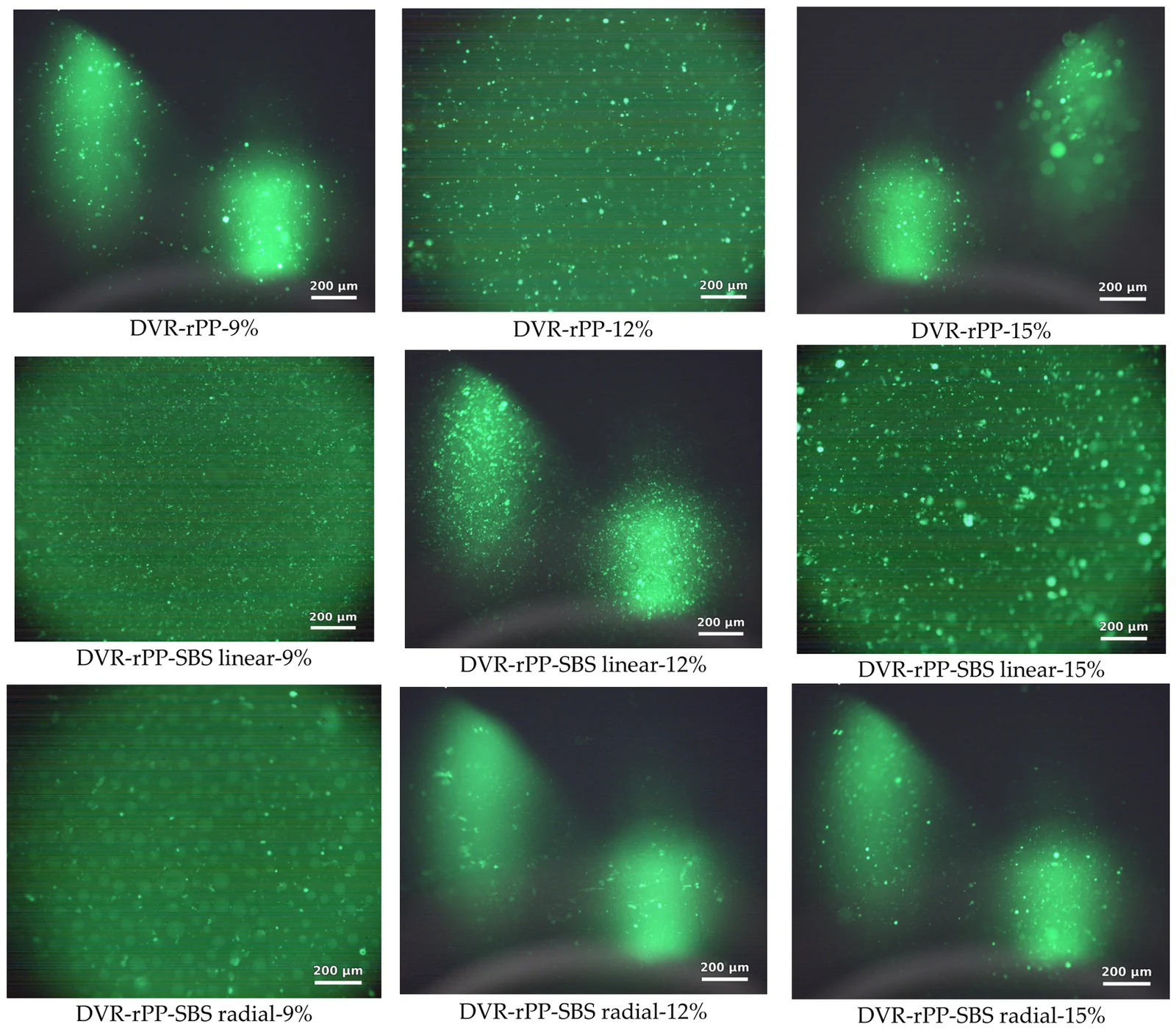
Fluorescence microscopy images of B70/100 bitumen modified with DVR-rPE, DVR-rPP, DVR-rPP-SBS-linear, and DVR-rPP-SBS-radial at 9, 12, and 15 wt.% modifier content.

At 9, 12, and 15 wt.% modifier content, fluorescence microscopy revealed distinct differences in particle size, dispersion, and concentration-dependent evolution among the four investigated DVR-based systems (Figure 11). In each case, the modifier phase appeared as fluorescent domains standing out against the darker bitumen matrix, consistent with the aromatic-rich, swollen character of the rubber/polymer-rich phase; the size, number, and spatial distribution of these fluorescent domains varied markedly with both modifier composition and concentration, providing qualitative insight into the differences in particle swelling and dispersion among the systems.

Among the four systems, DVR-rPE displayed the largest and most widely distributed fluorescent domains at all three concentrations, with isolated bright regions of markedly different size persisting from 9 to 15 wt.% without a clear, systematic trend of coarsening or refinement with increasing modifier content. DVR-rPP showed a fine, closely spaced particle pattern at 9 wt.%, but visibly coarsened with increasing concentration, developing several large, rounded, well-defined domains by 15 wt.%, consistent with progressive coalescence of the polymer-rich phase. DVR-rPP-SBS-linear displayed the smallest and most uniformly dispersed fluorescent particles among the four systems at every concentration examined, forming a fine, closely spaced pattern distributed evenly across the field that remained largely unchanged in character from 9 to 15 wt.%, with only a modest increase in the brightness and size of individual points at the highest content. DVR-rPP-SBS-radial, by contrast, showed the most pronounced concentration dependence: at 9 wt.%, the swollen phase appeared as a broad, diffusely fluorescent background with poorly defined boundaries, whereas by 15 wt.% this diffuse response had reorganized into two large, more sharply bordered domains with clearly resolvable internal granularity, indicating that a substantially more developed and spatially organized polymer–rubber network had formed at the higher modifier content.

These trends provided qualitative microstructural support for the mechanical and rheological results discussed in the preceding sections. The fine, densely and uniformly dispersed particle morphology of DVR-rPP-SBS-linear, essentially unchanged across the concentration range studied, was consistent with efficient formation of an interconnected, load-bearing polymer–rubber network already at low modifier content, in agreement with its superior elastic recovery, force ductility, and low-temperature flexibility reported above [1,53,54]. The coarse, widely distributed particle morphology of DVR-rPE, which did not show a clear concentration-dependent trend, indicated a less uniformly developed polymer–rubber phase, consistent with its comparatively limited elastic recovery and force ductility [55]. The concentration-dependent coarsening observed for DVR-rPP paralleled its intermediate and non-monotonic mechanical performance relative to the SBS-containing systems [1,9]. Finally, the marked reorganization of the DVR-rPP-SBS-radial morphology between 9 and 15 wt.%, from a diffuse background to well-defined domains, provided direct microstructural evidence for the concentration-dependent development of an effective elastomeric network previously inferred for this system from the force ductility and low-temperature flexibility results [1,54], rather than being based on those mechanical results alone. Because each image represents a single microscope field rather than a bulk average, these observations should be interpreted as qualitative, complementary evidence of particle swelling and dispersion rather than as a quantitative measure of network continuity; nonetheless, they support the general concept that swelling and partial dissolution of the polymer/rubber phase in the maltene fraction of bitumen underlies the property differences discussed in the preceding rheological and mechanical sections [1,13,48,53,54,55].

### 3.11. Mechanism of Interaction Between Partially Devulcanized Rubber and SBS-Modified Recycled Polymers

The interaction mechanism proposed in this section constituted a conceptual model, developed by combining the present FTIR, TGA, mechanical, and fluorescence-microscopy results with established descriptions of SBS- and rubber-modified bitumen from the literature. It was not directly proven by a single technique in this study, and the specific molecular-level steps (e.g., relative junction density of radial versus linear SBS) should be regarded as hypotheses generated by this model rather than as experimentally validated facts; direct techniques such as quantitative microscopy, small-angle scattering, or rheological network analysis would be required for confirmation. It should also be noted that the two SBS-containing modifiers (rPP-SBS-linear and rPP-SBS-radial) differ not only in SBS molecular architecture but also in DVR, rPP, and SBS content (Section 2.1); consequently, some of the performance differences attributed below to SBS architecture may also reflect these compositional differences, and the two effects cannot be fully separated with the present formulations.

The superior performance of the DVR-rPP-SBS-linear and DVR-rPP-SBS-radial systems was explained by the complex interactions occurring among bitumen, partially devulcanized rubber, recycled polypropylene, and SBS copolymer during high-temperature mixing.

Unlike conventional crumb rubber, partially devulcanized rubber contained a reduced density of sulfur cross-links while preserving a substantial fraction of the original polyisoprene and polybutadiene backbone structure. During blending at 180 °C, aromatic maltene fractions from the bitumen diffused into the DVR particles, causing significant swelling. This process increased the effective volume of the rubber phase and promoted interaction between the rubber particles and surrounding bitumen components. This is consistent with rheological studies on ground-tire-rubber-modified bitumens, where diffusion and swelling of the rubber phase were similarly shown to govern binder consistency development [13,48].

It should also be noted that the polymeric backbone of DVR was not compositionally uniform. Since crumb rubber feedstock is derived from end-of-life tires, its natural rubber (NR), SBR, and butadiene rubber (BR) content depends on the tire source: truck and heavy-vehicle tires typically yield DVR with a higher NR (polyisoprene) fraction, whereas passenger-car tires yield DVR with a higher SBR fraction. This source-dependent variability in rubber backbone composition is regarded as a factor influencing the degree of aromatic uptake, swelling, and entanglement capacity of DVR described below, and should be reported or controlled for when sourcing crumb rubber. [59,60] This is consistent with recent reviews of rubber devulcanization and recycling technologies, which similarly emphasize the compositional variability of tire-derived rubber feedstocks [51].

Simultaneously, SBS molecules became dispersed within the continuous bitumen phase. The polystyrene blocks tended to aggregate into rigid microdomains, while the polybutadiene segments remained flexible and compatible with both the rubber phase and aromatic constituents of bitumen. The polybutadiene chains of SBS were proposed to physically entangle with the partially devulcanized rubber chains, creating an interconnected elastomeric network throughout the binder. This is consistent with established descriptions of SBS-modified bitumen, in which performance enhancement is governed predominantly by polymer swelling, physical association, and phase morphology rather than by extensive formation of new covalent bonds [1,54].

**Figure 12:**
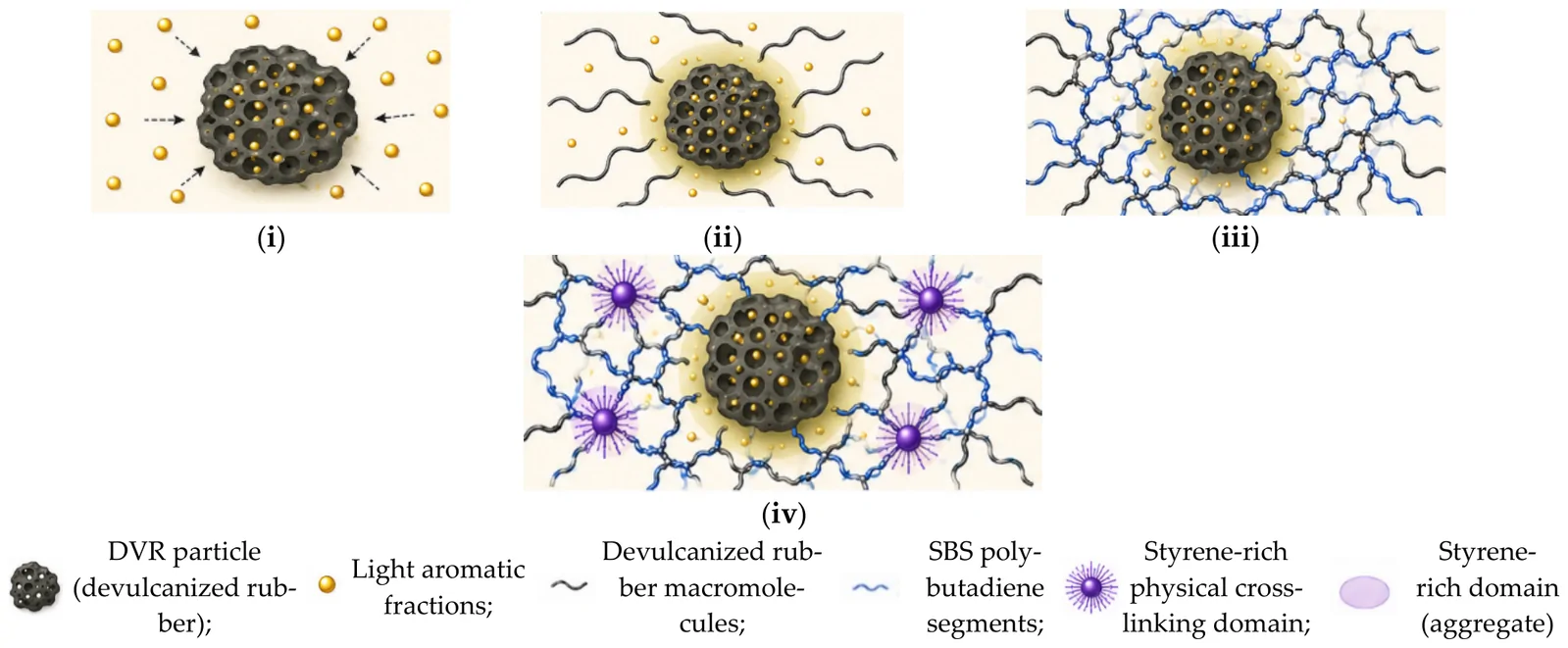
Main stages of the proposed reinforcement mechanism in the DVR-rPP-SBS-radial modified bitumen binder. For clarity, the schematic omits the recycled polypropylene (rPP) phase and focuses on the DVR–SBS interaction; the complete DVR–rPP–SBS structure is illustrated in Figure 10. (**i**) absorption of light aromatic fractions by DVR particles; (**ii**) swelling and softening of the rubber phase; (**iii**) physical entanglement between SBS polybutadiene segments and devulcanized rubber macromolecules; (**iv**) formation of styrene-rich physical cross-linking domains and development of a continuous three-dimensional polymer–rubber network.

The proposed reinforcement mechanism involves five principal stages, as shown in Figure 12.

Light aromatic molecules were proposed to diffuse from the bitumen phase and to be absorbed into the pores and micro-voids of DVR particles (Figure 12i). Absorbed aromatics were proposed to plasticize the rubber phase, causing it to swell and soften, which was expected to increase chain mobility and free volume (Figure 12ii). In the proposed sequence, flexible polybutadiene segments of SBS were hypothesized to interpenetrate and physically entangle with the devulcanized rubber macromolecules (Figure 12iii). Styrene blocks were proposed to aggregate together into rigid styrene-rich domains, which were hypothesized to act as physical cross-linking points (hard microdomains) (Figure 12iv). DVR particles were proposed to act as multifunctional junctions that, together with SBS and rubber chains, were expected to promote the development of a continuous three-dimensional polymer–rubber network throughout the bitumen binder.

Figure 12 depicted the DVR-rPP-SBS-radial system specifically (the rPP phase was omitted from the schematic for clarity): the star-shaped, multi-armed styrene-rich domains shown in stage (iv) were proposed to arise from the branched architecture of radial SBS, in which several styrene-block-ended polybutadiene arms radiate from a central coupling point and were hypothesized to allow a single SBS macromolecule to bridge and entangle with multiple DVR particles simultaneously. For the DVR-rPP-SBS-linear system, the same four stages (i)–(iv) applied, but stages (iii)–(iv) were proposed to proceed differently: because linear SBS consists of unbranched triblock chains, entanglement with DVR macromolecules was proposed to occur along extended, single-axis polybutadiene segments rather than through multiple radiating arms. Styrene-rich domains were likewise proposed to form as physical cross-linking points, but the resulting network was expected to be less dense and more chain-extended, potentially favoring continuity and low-temperature flexibility relative to the higher junction density hypothesized for radial SBS.

**Figure 13:**
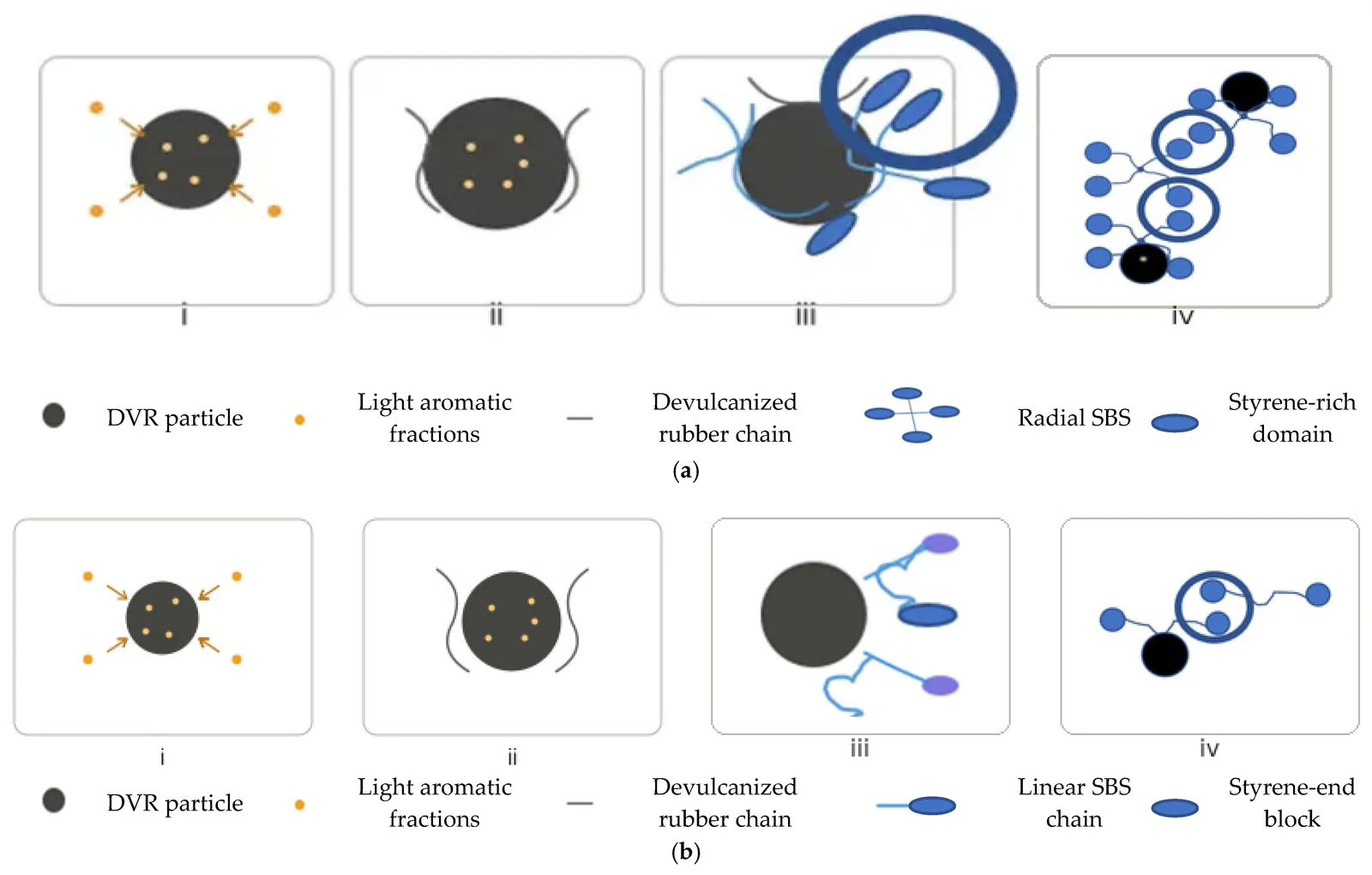
Schematic redrawing of the five-stage reinforcement mechanism, separated by SBS architecture: (**a**) DVR-rPP-SBS-radial system, showing star-shaped, multi-armed styrene-rich domains bridging several DVR particles; (**b**) DVR-rPP-SBS-linear system, showing elongated styrene end-block domains and chain-extended entanglement. Stages (**i**–**iv**) correspond to those described for Figure 12.

The two cases were illustrated separately in Figure 13a,b below.

This proposed network structure was expected to improve stress transfer, elasticity, and crack resistance in both architectures. The radial SBS architecture was hypothesized to be particularly effective at increasing junction density and rutting resistance, consistent with the multi-arm entanglement proposed in Figure 12; the linear SBS architecture was proposed to favor chain continuity and low-temperature flexibility, potentially at the cost of somewhat lower junction density.

The proposed interaction mechanism was consistent with previous studies demonstrating that the performance of SBS-modified and rubber-modified binders is primarily governed by the formation of physically cross-linked elastomeric structures and the development within the bitumen matrix [1,16,48,54,61,62]. This was consistent with the view that such networks form primarily through physical blending and swelling rather than chemical reaction, and that this network structure has been reported to underlie the improved viscoelastic balance and storage stability of SBS/rubber-modified binders in general (storage stability was not itself tested in the present study). The enhanced compatibility between partially devulcanized rubber and SBS observed in the present study was therefore proposed as a main contributing factor to the superior rheological and mechanical properties of the DVR-rPP-SBS-linear and DVR-rPP-SBS-radial systems.

## 4. Conclusions

This study evaluated the effect of partially devulcanized rubber (DVR) combined with four recycled-polymer systems—recycled polyethylene (rPE), recycled polypropylene (rPP), recycled polypropylene–linear SBS (rPP-SBS-linear), and recycled polypropylene–radial SBS (rPP-SBS-radial)—on the properties of B70/100 bitumen at 9, 12, and 15 wt.% modifier content. The main findings were as follows:

1. Increasing the modifier content from 9 to 15 wt.% reduced penetration and increased the softening point for all four systems; at 15 wt.%, DVR-rPP-SBS-radial produced the lowest penetration (42 × 0.1 mm) and the highest softening point, while DVR-rPP-SBS-linear achieved a total softening-point increase of approximately 28–30 °C relative to the base bitumen. Dynamic shear rheometry showed that all DVR-based modifiers extended the temperature at which |G*|/sin(δ) fell below the 1.00 kPa Superpave criterion; for DVR-rPP-SBS-linear at 15 wt.%, the rutting parameter increased by approximately 28–30 °C relative to the unmodified binder (≈58 °C).
2. DVR-rPP-SBS-linear exhibited the best overall balance of properties, with the highest elastic recovery (≈76% at 15 wt.%) and force ductility (≈8.6 J/cm^2^ at 15 wt.%) and the lowest Fraass breaking point (−41 °C, a 25 °C reduction relative to the base bitumen).
3. DVR-rPP-SBS-radial reached the highest softening point at 15 wt.% and a Fraass breaking point (−37 °C), only a few degrees above that of DVR-rPP-SBS-linear, confirming that both SBS-containing systems provided the most effective combination of high- and low-temperature performance.
4. Between the two SBS-free systems, DVR-rPP showed the least improvement in penetration reduction and low-temperature flexibility, whereas DVR-rPE exhibited the lowest elastic recovery (≈41% at 15 wt.%) and force ductility, indicating the limitations of recycled-polyolefin-only modification.
5. FTIR and GF indicated that partial devulcanization preserved the elastomeric backbone of DVR; the resulting polymer–rubber network was proposed as the mechanism underlying the improved high- and low-temperature performance of the SBS-containing systems.
6. Overall, DVR-rPP-SBS-linear was identified as the most promising sustainable modifier for producing high-performance bituminous binders from waste-derived materials. Further studies on MSCR, fatigue and low-temperature rheological characterization, aging resistance, storage stability, and asphalt-mixture and field-performance evaluation are recommended to confirm its long-term applicability in pavement engineering.

## Figures and Tables

**Table 1:** Physicochemical properties of petroleum road viscous bitumen (grade B70/100).

Bitumen Properties	Actual Value	Test Method	Specification Requirement (EN 12591 [42,43,44])
Penetration at 25 °C, 0.1 mm	87.2	EN 1426:2015 [42]	70–100
Softening point °C	46.6	EN 1427:2015 [43]	43–51
Fraass breaking point °C	−16	EN 12593:2015 [44]	≤−10

**Table 2:** Devulcanized rubber samples.

Samples	DVR-rPE	DVR-rPP	DVR-rPP-SBS-Linear	DVR-rPP-SBS Radial
Photo of samples	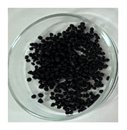	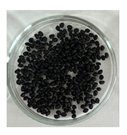	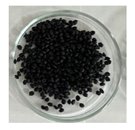	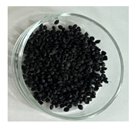

## Data Availability

The original contributions presented in this study are included in the article. Further inquiries can be directed to the corresponding author.
